# The importance of integrated therapies on cancer: Silibinin, an old and new molecule

**DOI:** 10.18632/oncotarget.28587

**Published:** 2024-05-23

**Authors:** Elisa Roca, Giuseppe Colloca, Fiorella Lombardo, Andrea Bellieni, Alessandra Cucinella, Giorgio Madonia, Licia Martinelli, Maria Elisa Damiani, Ilaria Zampieri, Antonio Santo

**Affiliations:** ^1^Oncologia Toracica - Lung Unit, Ospedale P. Pederzoli - Via Monte Baldo, Peschiera del Garda (VR), Italy; ^2^Dipartimento di Scienze dell’invecchiamento, Neurologiche, Ortopediche e della testa-collo, Fondazione Policlinico Universitario “A. Gemelli” IRCCS, Rome, Italy

**Keywords:** silibinin, anti-inflammatory, inflammation, toxicity, integrated therapy

## Abstract

In the landscape of cancer treatments, the efficacy of coadjuvant molecules remains a focus of attention for clinical research with the aim of reducing toxicity and achieving better outcomes.

Most of the pathogenetic processes causing tumour development, neoplastic progression, ageing, and increased toxicity involve inflammation. Inflammatory mechanisms can progress through a variety of molecular patterns. As is well known, the ageing process is determined by pathological pathways very similar and often parallel to those that cause cancer development. Among these complex mechanisms, inflammation is currently much studied and is often referred to in the geriatric field as ‘inflammaging’.

In this context, treatments active in the management of inflammatory mechanisms could play a role as adjuvants to standard therapies.

Among these emerging molecules, Silibinin has demonstrated its anti-inflammatory properties in different neoplastic types, also in combination with chemotherapeutic agents.

Moreover, this molecule could represent a breakthrough in the management of age-related processes.

Thus, Silibinin could be a valuable adjuvant to reduce drug-related toxicity and increase therapeutic potential.

For this reason, the main aim of this review is to collect and analyse data presented in the literature on the use of Silibinin, to better understand the mechanisms of the functioning of this molecule and its possible therapeutic role.

## INTRODUCTION

Inflammation underlies many disease processes, and among these, ageing and the development of neoplasms would seem to have similarities and parallels underlying an important inflammatory substrate.

**Figure 1:**
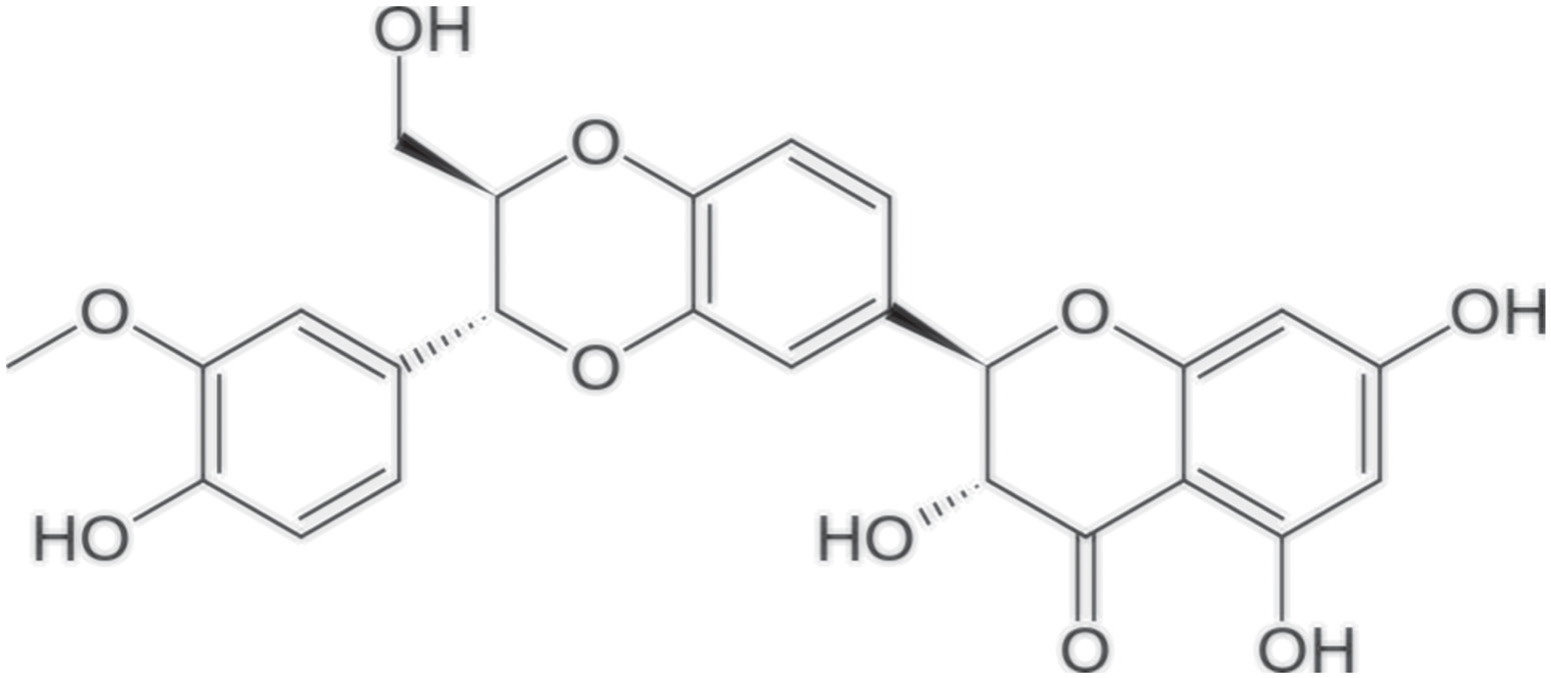
Molecular structure of Silibinin.

Silibinin was isolated from the seeds of milk thistle (Silybum marianum) and it a polyphenolic flavonolignan [1] used to date as a treatment for hepatopathies and for different exchange mechanisms; it is also a molecule with inflammatory, antioxidant and anti-fibrotic properties [1] (Figure 1).

Furthermore, there are promising data on the application of Silibinin as an anti-cancer coadjuvant in various types of neoplasia [2].

In this regard, the antitumour characteristics of Silibinin have been documented both *in vivo* and *in vitro*.

In particular, this molecule seems to play a role in inhibiting proliferation, blocking metastasis and tumour invasion, and inhibiting angiogenesis.

Furthermore, Silibinin could be useful as an inducer of programmed death (apoptosis) and autophagy.

Finally, studies show a role for Silibinin in cell cycle arrest and in the inhibition of certain signalling pathways such as MAPK, STAT3, Notch-1, ERK and Akt.

For this reason, Silibinin could be used in combination with antiblastic drugs to achieve a synergistic effect and improve the outcomes of neoplastic patients.

Since several studies show that Silibinin is implicated at some critical points in the processes of ageing and ‘inflammation’, this treatment could counteract ‘inflammation’ mechanisms above all in older adults [2].

## ROLE OF SILIBININ IN DIFFERENT CANCER’S TYPE

Studies investigating the efficacy of Silibinin as an anticancer agent are promising [2–4]; in particular, the most interesting data concern its effectiveness as an adjuvant in the treatment of gliomas.

Gliomas are the most common brain tumors, which maintain a poor prognosis, despite the aggressive therapeutic strategies (surgery, radiotherapy, chemotherapy) that are implemented to increase the survival of patients affected by this neoplasm [5].

To reduce the side effects of these treatments and to increase their effectiveness, therapeutic strategies also based on natural compounds are currently being taken into consideration [6].

In this context, research regarding Silibinin has shown interesting results regarding its ability to inhibit cell proliferation [7] and its role in sensitizing glioma cells to apoptosis [8].

The promising results of these studies suggest that Silibinin could inhibit cell proliferation by increasing the intracellular Ca2+ concentration. This increase in intracellular Ca2+ appears to have a critical role in regulating cell death mediated by a calpain-dependent pathway and in inducing the generation of ROS. Several studies also show that Silibinin is able to generate ROS through a CA2+ -dependent mechanism by the respiratory chain in the inner mitochondrial membrane [9] or through direct stimulation of the generation of mitochondrial ROS [10].

Furthermore, this milk thistle-derived molecule can induce programmed cell death by activating mitogen-activated protein kinases (MAPKs) through the generation of ROS.

Moreover, Silibinin is able to activate (via ROS) and inhibit (via antioxidant N-acetylcysteine (NAC)) the extracellular signal-regulated kinase (ERK), the p38 kinase and the N-terminal c-Jun kinase (JNK) in time mode [11]. This suggests that MAPK activation is likely involved in Silibinin-induced cell death.

Another mechanism believed to be secondary to the action of Silibinin is the inhibition of glioma cell migration.

Regarding *in vivo* antineoplastic effects, some authors have reported data on Silibinin administered orally in mice with gliomas: these data demonstrated a decrease in glioma growth *in vivo*, thanks to the administration of this molecule.

In particular, the regression of tumor volume could be induced by Silibinin, leading cells to programmed death through a caspase-dependent mechanism, involving the Ca2+/ROS/MAPK pathways, [5, 12].

In addition, Silibinin appears to play a role in cell migration through mitochondrial fusion and inhibition of ROS [13].

Some studies have also shown the ability of Silibinin to promote the epithelial-mesenchymal transition: this transformation could be due to the decrease in the expression of proteins linked to migration (for example metalloproteinases 2 and 9) or to the increase in the expression of other biomarkers, including E-cadherin.

The discovery of the epidermal growth factor receptor (EGFR) and the possibility of using it as a therapeutic target has changed the natural history of many patients suffering from cancer.

In the context of gliomas, rat cell lines expressing EGFR were tested to evaluate the ability of Silibinin to inhibit mitogenic signaling, regulate cell survival and alter the cell cycle [14, 15]. The researchers noted that EGRF-positive cells demonstrated cytotoxicity in response to Silibinin, on the other hand, a lack of toxicity was observed in cell lines without EGFR sequences. Moreover Silibinin seems to be able to inhibit the binding of EGF to the EGFR resulting in cytotoxicity and with unknown effects on the dimerization of the receptor.

## SILIBININ AS STAT3 INHIBITOR

Silibinin has been shown to be both *in vitro* and *in vivo*, a physiological down-regulator of STAT3, a protein which is part of “signal transducer and activator of transcription” family (STAT) activity [16]. This molecule appears to have a role in reducing the incidence of main chemotherapy-related toxicities, such as nephrotoxicity, neurotoxicity and cardiotoxicity in pre-clinical models, as well as in preventing drug resistance mechanisms.

Members of STAT family share a similar functional structure: they are cytoplasmic proteins, containing Src homology 2 (SH2) domains. Being responsive to different levels of cell growth factors and cytokines they work as transcriptional factors [17]. Currently STAT3 is the most known member of STAT family, since several studies have shown that altered regulation of this protein may underlie loss of cell cycle control, uncontrolled cell survival, carcinogenesis, and metastatic dissemination and immunoresistance processes. Accordingly, this protein has been largely considered as a potential therapeutic target in different tumor types, leading to the development of a new class of antineoplastic drugs, STAT3 inhibitors, classified as indirect or direct based on their mechanism of action [17, 18].

Given its modulatory function on STAT3, Silibinin is under evaluation in the oncological research field both as monotherapy and in combination with other available therapeutic regimens [19].

## BRAIN METASTASIS GROWTH

Among intracranial tumors, metastases are the most common form of central nervous system (CNS) involvement in adults (more than 50%). The process of brain metastasis appears to be the most challenging for tumor cells, due to the presence of the blood-brain barrier (BBB) and the distinctive microenvironment [20]. The metastatic potential of cancer cells increase as they acquire the ability to escape natural defense mechanisms, interact with various cell types in different cellular microenvironments, affecting their activity in a pro-metastatic way, and lastly promoting their own survival [21, 22]. Within this specific microenvironment, astrocytes and microglia cells seem to play a critical role in the process of promoting brain metastatization. In fact, astrocytes are CNS cells able to react to tissue damage by the activation of a morphological and transcriptomic reactive state (Reactive astrocytes- Ras), which has been demonstrated to affect the course of several neurological diseases including secondary SNC lesions [23]. This reactive state also appears to be mediated by STAT3 activation, resulting in a pro-metastatic environment [24, 25]. Furthermore, STAT3 hyperactivation in reactive astrocytic phenotype (RAs) could act negatively on the activation of both innate and acquired immune response and in particular on CD8+ lymphocytes, of which also brain metastases are often infiltrated. Activated STAT3 RAs cells could therefore act by reducing the tumor infiltrate and creating an immunosuppressive microenvironment [26].

The importance of STAT3 activation for CNS colonization, microenvironment changes, and the potential utility of administering anti STAT 3 drugs also in combination with Immune Checkpoint Inhibitors (ICIs) is supported by the fact that Silibinin is able to cross the BBB and reduce STAT3 expression in the tumor microenvironment. It was used in an RCT test to test the overall survival from brain metastasis diagnosis compared to a control cohort.

## COADJUVANT MECHANISM OF SILIBININ

### Protector on chemotherapy-induced toxicity

Most antiblastic drugs are metabolized/excreted by the kidney, potentially resulting in dose-related renal toxicity, as acute or chronic kidney injury and electrolyte disorders. Sometimes renal damage may be irreversible [2, 27, 28]. Several studies have demonstrated a protective role of Silibinin toward renal function when administered together with nephrotoxic drugs such as cisplatin and vinorelbine [29] (Table 1).

**Table 1:** Fields of application and mechanisms of Silibinin

CANCER MECHANISMS	⇓ Ca2+/ROS/MAPK ⇓ STAT3 ⇑ Sensibility to apoptosis ⇓ Tumor cell proliferation ⇓ Metastasis growth ⇓ Resistance to chemo/radio/target therapies
NEUROPATHY (CIPN)	⇓ Microtubule disruption ⇓ Oxidative stress ⇓ Mitochondrial damage ⇓ Altered ion channel activity ⇓ DNA Damage ⇓ Immunological processes
INFLAMMATION	⇓ IL-6 ⇓ ROS
AGING	⇓ Oxidative stress ⇓ Mitochondrial Dysfunction ⇓ Shortened Telomeres ⇓ DNA Damage ⇓ Cell Senescence

### Silibinin in reversing resistance to chemotherapy

STAT pathway activation has been associated with primary chemoresistance and the development of secondary chemoresistance [30]. Based on this evidence, it is expected that inactivation of STAT3 may play a synergistic role by potentiating the effect of chemotherapeutic drugs [31].

Among these, the greatest evidence in terms of synergistic efficacy was obtained combining Silibinin with paclitaxel: known as a microtubule stabilizer, paclitaxel has been shown to reduce STAT3 protein activity (Tyr705) and to inhibit the expression of STAT3-target genes via a negative feedback loop [32]. Further evidences have been obtained combining Silibinin with Arsenic Trioxide (ATO) in human glioblastoma cell line, in which a prevalence of apoptotic phenomena and reduced invasive capacity were observed [33].

### Silibinin in reversing resistance to targeted therapies

The activation of STAT3 is among the pathways proposed to be involved in the development of drug resistance in oncogene-addicted tumor cells, although the specific mechanism is still unclear: STAT3 could be activated to prevent apoptosis and sustain cell viability but it may also be activated early in a cancer-cell sub-population, maybe immediately after drug exposure [34].

In this setting, elevated levels of pSTAT3 have been associated to poor prognosis and lower overall survival in patients affected by hepatocellular carcinoma, where this molecule plays a pivotal role in inflammation, and in sustaining survival, proliferation, and invasion capability of malignant cells [35]. Numerous studies have also demonstrated the essential role of STAT3 in the development of drug resistance toward epidermal growth factor receptor (EGFR) tyrosine kinase inhibitors (TKIs): high levels of STAT3 have been recently reported to predict worse progression-free survival.in patients affected by Non-Small Cell Lung Cancer (NSCLC) harboring EGFR activating mutations treated with this class of drugs [36, 37]. Interestingly, Silibinin have been shown to be able to overcome such resistance towards two different TKI, erlotinib and gefitinib, both *in vivo* and *in vitro*, although the ability of Silibinin to revert such resistance acting through the pSTAT pathtway inhibition is yet to be confirmed [38–40].

### Silibinin in reversing resistance to radiotherapy

Radio-resistance is one of the main limitations to the efficacy of radiation therapy and may be induced by the use of fractionated radiation. At a cellular level, this has been associated to EMT: cancer cells surviving to ionizing radiations often feature EMT-like phenotype, that appears to be essential for the development of radio-resistance [41]. Radiation-resistant cancer cells have been found to feature high levels of nuclear expression of STAT3 [42], and some studies suggested that the inhibition of this pathway can reverse the EMT process, thus overcoming radio resistance [43–45]. Based on these data, the combination of ionizing radiation with Silibilin may represent a possible strategy to overcome radio resistance by inhibiting STAT3 signaling and therefore EM transition [46].

## INFLAMMAGING

The process of aging is the combination of various factors, involving a variety of environmental, stochastic, and genetic-epigenetic events. One of the pivotal mechanisms involved is inflammation, that usually contributes to aging process through the development of the so-called “inflammaging”: a chronic, low-grade inflammation status [47, 48].

Various organs and tissues, including bone and muscle and adipose tissue, are able to produce pro-inflammatory compounds, and, as such, inflammaging appears to be a systemic, multifactor and multiorgan condition, determined by a complex balance between pro- and anti-inflammatory factors. It can be considered among the main factors in the pathogenesis of various age-related diseases, such as atherosclerosis, type II diabetes, as well as of many chronic conditions including sarcopenia [49], osteoporosis [50], frailty and disability [51]. Overall, this inflammatory process can contribute to increase the risk of multimorbidity and disease susceptibility, and to impair the ability to appropriately respond to stress as well as to treatments, ultimately favoring the development of geriatric syndromes, disabilities, and death.

Multiple processes, both at a cellular and molecular level, have been taken in account to explain inflammaging, among them inflammasome activation, dysbiosis, cellular senescence, mitochondrial dysfunction, defective autophagy and mitophagy, ubiquitin-proteasome system dysregulation, activation of the DNA damage response [52], and chronic activation of the innate immune system [48].

Multiple epidemiological and biodemographic studies have shown that inflammation biomarkers can be robust predictors of morbidity and mortality in the elderly [53, 54]. Among the multiple factors involved, interleukin-6 (IL-6), with its complex pro-and anti-inflammatory functions, deserves a primary role, and has been associated to multiple conditions and age-related disease as well as to mortality in the elderly [55].

Some authors have also suggested that inflammaging could be considered in some way a form of autoimmune condition in which the distinction between self and non-self structures get increasingly blurred with age, and that this process could be caused by the malfunction of some of the various mechanisms appointed to dispose of senescent or apoptotic cells [51]. Indeed, the process may be triggered by the oxidative stress caused by the accumulation of free radical within a cell can activate a network of distress sensors (Nlrp3 inflammasome) thus activating the immune response and the production of pro-inflammatory cytokines such as IL-1beta and IL-8. Hence, Inflammaging may be triggered by the slow accumulation with age of damaged macromolecules and cells, that lead with time to chronic stress.

The same aging process that affects the human body’s microbial constituents (i.e., gut microbiota), altering their physiological functions (i.e. elimination of toxic substances, production of important metabolites and obstacle to the growth of microorganisms harmful to the organism), is responsible for a state of chronic low-grade inflammation mediated by binding to pattern recognition receptors (PPR), the activation of peroxidation processes, membrane rupture and release of cellular components into the extracellular space and the triggering of autoimmune reactions.

Standard cell components [56], considered part of the cellular self, usually do not stimulate immune and inflammatory reactions. However, when they are misplaced and occur outside of their standard physiological location, they could sense and bind pattern recognition receptors (PPRs) inducing an inflammatory response.

The misplacement of self-molecules appears to increase with age, supporting the hypothesis that inflammaging is fueled by increased exposure of cell components. Given that mitochondria are reminiscent of their ancestral bacterial origin, their parts share with bacteria the ability to bind to PRRs leading to an inflammatory response.

The inflammatory phenotype originates inside cells with different organelles’ contributions, including the nucleus, and various mechanisms, such as telomere attrition, DNA damage response (DDR), mitochondrial dysfunction, proteasome/lysosome alteration, inflammasome activation, and endoplasmic reticulum (ER) stress. Supporting this idea, human longevity is characterized by the preserved function of proteasomes [57] and autophagy (both mechanisms involved to prevent the accumulation of cellular components): the activity of the ubiquitin-proteasome system and autophagy have also been associated with the rate of aging [58, 59], and a higher expression of the immunoproteasome is a sign of neuroinflammation, as found in the brains of patients with Alzheimer Disease (AD) but not of healthy older subjects [60].

These misplaced molecules are not confined within the cell and actively or passively leave them, being taken up by other distal cells and triggering a feed-forward propagation–amplification cycle of inflammation and inflammaging in distant cells by traveling via the circulatory and lymphatic systems. This suggests that inflammaging is propagated by the secretion of damaged cellular components produced by compromised, stressed, or senescent cells and organelles during a pathological event [61], transmitted in a paracrine fashion by activation of IL-1 signaling [62]. Local propagation can be significant in pathologies such as cancer in older subjects, where systemic inflammaging occurs [63]. Moreover, systemic inflammation can accelerate aging via reactive oxygen species (ROS)-mediated exacerbation of telomere dysfunction and cell senescence in the absence of any other genetic or environmental factors [64].

There is, therefore, excellent attention today towards all those products capable of reducing these inflammatory mechanisms at the base of aging and all those changes associated with it (Table 1).

### Peripheral neuropathy

Inflammation is also the basis of chemotherapy-induced painful peripheral neuropathy (CIPN).

In particular, chemotherapeutics damage the nervous system through mechanisms involving microtubule disruption, oxidative stress, mitochondrial damage, altered ion channel activity, DNA damage, immunological processes, and neuroinflammation [65]. which leading to the CIPN.

CIPN is a predominantly sensory neuropathy as a typical “glove and stocking” neuropathy; it is characterized by high prevalence among cancer patients negatively impacting their quality of life and it varies in intensity and duration usually emerging weeks or months after chemotherapy completion.

For these reasons, chemotherapy-induced painful neuropathy (CIPN) is considered a significant dose-limiting side effect of several chemotherapeutic agents limiting therapeutic options for patients.

Six main agent groups can result in CIPN development: platinum-based antineoplastics (particularly oxaliplatin and cisplatin), vinca alkaloids, epothilones, taxanes, proteasome inhibitors, and immunomodulatory drugs.

Data suggest that, as a natural antioxidant compound, the administration of Silibinin in combination with oxaliplatin, the best known among chemotherapeutics for inducing CIPN through oxidative stress), can prevent oxidative damage reducing oxaliplatin-dependent pain neuropathy. Accordingly, Silibinin could be a valid therapeutic option for CIPN [66] (Table 1).

## CONCLUSIONS AND POTENTIAL NEW FIELDS

Inflammation is a critical process in tumor progression as demonstrated by the fact that cytokines released in chronic inflammatory conditions provide a microenvironment favourable to tumor growth and metastasis [67]. In particular, mitochondrial damage it would seem plays an essential role in inflammation as it is a prerequisite for the assembly and activation of inflammasome through oxidized mitochondrial DNA (mtDNA) released in a ROS-dependent manner [68, 69]. On the basis of these considerations, data suggest that Silibinin and NAC administration, limiting ROS generation, can decrease the production of ox-mtDNA in human triple-negative breast cancer cells, [13, 70, 71].

This consideration could be the starting point to study whether Silibinin could contrast tumor progression and aging and inflammaging through molecular and cellular mechanisms until now not completely clarified.

Furthermore, data suggest that the inhibition of STAT3 mediated by Silibilin, could be considered an interesting therapeutic approach also for patients with brain metastases. Indeed, this setting of patients maintains a poor prognosis with limited treatment options, therefore, further investigations on the role of Silibilin in this setting of patient could offer better outcomes and survival benefit.

With the aim of confirming this hypothesis a randomised phase 2 study is investigating the preventive role of Silibinin against placebo in patients who have undergone complete resection of a brain metastasis from NSCLC and breast cancers (NCT05689619).
